# Intersection of nanomaterials and organoids technology in biomedicine

**DOI:** 10.3389/fimmu.2023.1172262

**Published:** 2023-04-28

**Authors:** Chen Shen, Zi-jian Zhang, Xiao-xue Li, Yun-peng Huang, Yong-xiang Wang, Hui Zhou, Li Xiong, Yu Wen, Heng Zou, Zhong-tao Liu

**Affiliations:** ^1^Department of General Surgery, Second Xiangya Hospital, Central South University, Changsha, Hunan, China; ^2^Department of Obstetrics and Gynecology, Second Xiangya Hospital, Central South University, Changsha, Hunan, China

**Keywords:** nanomaterials, organoids, 3D culture, nanoparticles, biomedicine

## Abstract

Organoids are stem cell-derived, self-organizing, 3D structures. Compared to the conventional 2D cell culture method, 3D cultured organoids contain a variety of cell types that can form functional “micro-organs” and can be used to simulate the occurrence process and physiological pathological state of organ tissues more effectively. Nanomaterials (NMs) are becoming indispensable in the development of novel organoids. Understanding the application of nanomaterials in organoid construction can, therefore, provide researchers with ideas for the development of novel organoids. Here, we discuss the application status of NMs in various organoid culture systems and the research direction of NMs combined with organoids in the biomedical field.

## 1 Introduction

Organoids are 3D structures grown from stem cells that consist of self-organizing organ-specific cell types shaped by cell classification and spatially constrained cell lines (1). These stem cells may be embryonic stem cells (iPSc) derived or adult stem cells (aSCs). During development, organoid formation recapitulates two primary processes of self-organization: cell classification and spatially restricted cell line typing. Human organoids reproduce developmental patterns, thereby replicating the structure and physiology of specific tissue types, making it possible to accurately study human disease and supplant animal experiments.

James Rheinwald and Howard Green first described the long-term culture of normal human epidermal cells in 1975 by combining freshly isolated keratinocytes with multi-mouse 3T3 fibroblasts and isolating keratinocytes without viable fibroblasts (2). However, this method of cell culture resembles two-dimensional plane culture. In 2009, Hans Clevers et al., successfully inoculated adult Lgr5(+) intestinal stem cells from mouse intestines in matrigel and added Wnt pathway agonist R-spondin, TGF-β inhibitor Noggin, epidermal growth factor, and other stem cell growth factors to cultivate a three-dimensional structure with crypt-like and villiform-like epithelial regions (small-intestinal organoids) (3). Accordingly, it comprised the first organoid to meet the modern definition, ushering in a “new era” in the development of organoid technology. Since then, numerous organoids have emerged, including those of the brain (4), stomach (5), colon (3), liver (6), kidney (7), heart (8), pancreas (9), prostate (10), and numerous other tissues and organs, as well as organoids of various cancerous tissues (11–13). Increased interest in tissue engineering, disease modeling, precision medicine, drug screening, and immunotherapy has resulted from the development of organoid culture (14). The rapid development of organoid technology has introduced novel concepts to the study of a variety of diseases. Using the intraductal transplant organoid (IGO) model, Tuveson et al., developed classical subtypes of pancreatic ductal adenocarcinoma (PDAC) in order to study subtype-dependent therapies that provide a deeper understanding of the genetic and epigenetic dynamics of PDAC (15). Park et al., utilized human colon organoids to evaluate the toxicity induced by SiO2 and TiO2 nanoparticles and to increase the expression of the apoptosis marker Bax/Bcl-2. This study demonstrated a difference in toxicity between 2D models and 3D organoid cultures, highlighting the significance of organoids in drug screening (16). In addition, the organ-on-a-chip, which combines microfluidics and organoid technology, enables precise regulation of the organoid microenvironment as well as precise simulation of multi tissue crosstalk with low heterogeneity (17).

As an emerging 3D physiological model, organoids possess the potential to change the methodology of research in the medical field. However, due to technical limitations, at present, various organoids still have quite a few defects (18). For example, we still cannot very precisely control organoid size, shape, proportion of cellular composition (6). More importantly, researchers cannot control the growth and function of organoids matching (19), which produce internal tissue necrosis after growing to a certain scale. The key to addressing these issues is the development of culture systems. The application of nanomaterials brings new ideas to solve these problems. Nanomaterials are a kind of materials ranging from 1-100 nm (20). Nanomaterials are materials between 1–100 nm in size (20). The use of nanomaterials has altered numerous fields, such as medicine, agriculture, manufacturing, electronic technology (21–23), and their unique optical, magnetic, and electrical properties render them irreplaceable in terms of their application potential. Accordingly, nanomaterials play an increasingly vital role in the field of biomedicine, and also significantly enhance and expand the research value of organoids (24). Mo et al. developed electro spun nanofibers prepared based on P (LLA-CL) copolymer and cultured smooth muscle cell (SMCs) and endothelial cells (ECS) as scaffolds and showed that these cells proliferated well on the nanofibrous scaffolds (25). This study suggests that the nanoscale culture environment will have an impact on the behavior and function of cells. In addition, nanomaterials may promote angiogenic effects in culture systems (26), which is helpful for addressing the problem of imbalance in organoid growth and function. Therefore, we have sufficient reasons to conclude that the application of nanomaterials constructed culture system has a positive effect on 3D culture of organoids.

**Figure 1:**
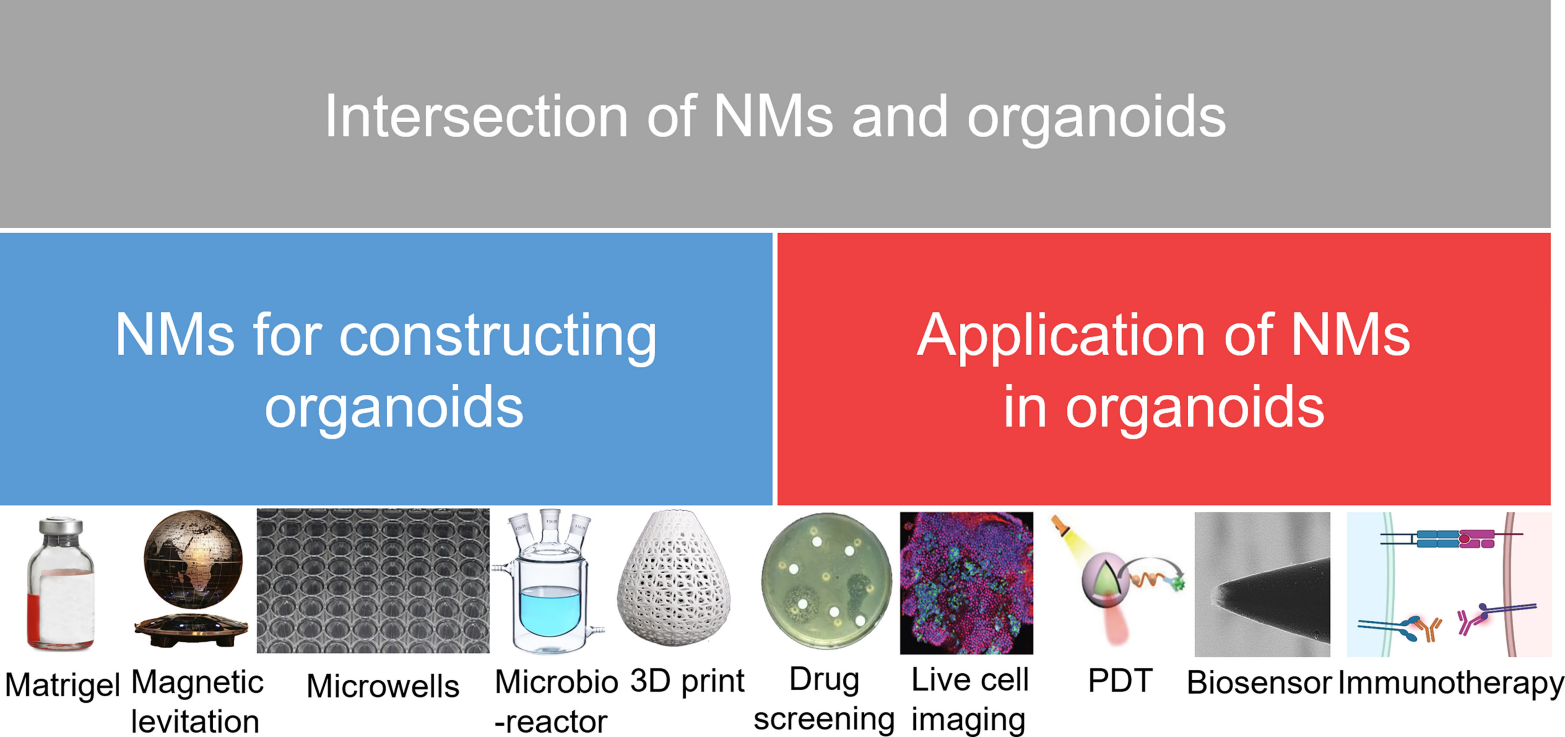
Research directions in the development of NMs and organoids. NMs, nanomaterials; PDT, photodynamic therapy.

The relationship between nanomaterials and organoids has been discussed in many excellent reviews (27–31). However, no review has yet been published that focuses on how nanomaterials are extensively involved in organoid construction. Consequently, this review will focus on the application status and future prospects of nanomaterials in the field of organoids, as well as the state of the frontier research for the combined application of organoids and nanomaterials in biomedicine (**Figure 1**).

## 2 Use nanomaterials to assist in the construction of organoid 3D culture systems

The advancement of nanomaterial technology has inspired the creation of new organoids, and nanomaterials can aid in the construction of organoids in numerous ways. Organoids are created using two types of stem cells: (1) pluripotent embryonic stem cells (ES), and their synthetic induced pluripotent stem cell (iPS) counterparts, and (2) adult organ-restricted stem cells (aSCs) (32). Conventional organoid culture systems also necessitate 3D solid extracellular matrices containing laminin, collagen, and other growth factors. To encourage organoid differentiation, it is also necessary to add growth-stimulating factors. Different organoids require distinct construction steps and the addition of a growth stimulant. In addition to conventional organoids based on a 3D solid external matrix, various novel organoid-building techniques have been developed in recent years (33). For example, Wiedenmann et al., designed a microwell chip to generate defined 3D aggregates of pancreatic progenitor cells derived from human induced pluripotent stem cells (hiPSCs) and then induced their differentiation into pancreatic duct-like organoids (34). Additionally, Ferreira et al., developed saliva-secreting organoids/microglands using a novel scaffold/substrate-free culture system known as magnetic 3D suspension (M3DL), which assembles and levitates magnetized primary SG-derived cells (SGDCs) so that they can generate their own extracellular matrix (35). Thus, nanomaterials have not only been utilized in conventional methods based on a 3D solid outer matrix, but they also play a significant role in a few novel organoid culture strategies. Accordingly, this review summarizes the key role of nanomaterials in organoid construction with respect to the aforementioned factors.

### 2.1 The use of nanomaterials to alter the properties of the extracellular matrix

Most existing organoid 3D culture systems were developed primarily on the basis of Matrigel (33). Matrigel, a substance secreted by Engelbreth-Holm-Swarm mouse sarcoma cells (36) contains laminin, type IV collagen, and heparin sulfate proteoglycan 6,7, which promotes cell adhesion, survival, andorganoid formation (37). As a traditional organoid culture system, Matrigel seem to gradually fail to meet the needs of researchers to develop better organoids. Many kinds of hydrogel replacement materials are being used for the development of organoids (38).

Using the properties of nanomaterials, it is possible to modify certain properties of matrix gum in order to create the desired organoid model. For instance, in the study by Bao et al., carbon nanotubes (CNTs) were used to regulate extracellular matrix (ECM) viscosity and intracellular energy metabolism. In addition, CNTs reduced the hardness of the extracellular matrix by reducing elasticity and increasing viscosity. Moreover, carbon nanotubes modified the metabolic profile of intestinal organoids and increased mitochondrial activity, respiration, and nutrient absorption. These synergistic mechanisms promote the proliferation and differentiation of intestinal organoids. This hints at the possibility of CNTs as biomaterials for intestinal tissue engineering (39). Purwada et al., introduced a B-cell follicular organoid composed of nanocomposite biomaterials, on which researchers continuously provided an extracellular matrix (ECM) and intercellular signals to naïve B cells, thereby accelerating the induction of germinal center (GC) response. The silicate nanoparticles complexed with gelatin utilized in this study comprised ultrathin nanomaterials with a high level of anisotropy and functionality. These nanoparticles have great potential in regenerative medicine and drug delivery (40). Luo et al., synthesized bone-forming peptide-1 (BFP-1)-loaded mesoporous silica nanoparticles (pep@MSNs) incorporating adhesion peptides that contained arginine-glycine-aspartate (RGD) domains, which modified alginate hydrogel (RA) system (pep@MSNs-RA) to promote the activity and sequential stimulation of bone differentiation in human mesenchymal stem cells (hMSCs). This ensured enhanced hMSC survival and proliferation in adhesion peptide-modified hydrogels. After the phase of proliferation, BFP-1 then induced bone differentiation of hMSCs derived from pep@MSNs. Additionally, BFP-1was self-captured by an additional cellular peptide cross-linking network formed by receptor-bound ligands (RGDs) on the cell surface, resulting in long-term sustained bone stimulation of hMSCs. The results demonstrated that independent and sequential stimulation of the proliferation and bone differentiation stages synergistically increased hMSC survival, amplification, and osteogenesis compared to stimulation alone or simultaneously (41). Thus, nanomaterials can create new matrigel culture systems and novel organoid models.

### 2.2 Develop novel microwells with nanomaterials to culture organoids

Low throughput (approximately 4 organoids per square millimeter) and poor repeatability are disadvantages of conventional matrigel-based organoids. Microwells are widely used to capture single cells and are simple to fabricate, convenient to operate, and high-throughput (42).

Thus, additional optimization is required in terms of scale, morphogenetic stability, and compatibility with high-throughput phenotypic analysis. Accordingly, organoid technology based on micropores was developed (43). Shin et al., utilized a microporous array-based 3D culture system with a polycaprolactone (PCL) nanofiber bottom and a polyethylene glycol (PEG) hydrogel wall for efficient bioengineering of human salivary gland organoids that can readily generate uniformly sized 3D organoids. In comparison to Matrigel and nanofiber scaffold cultures, the results demonstrated greater efficacy. The novel aspects of this study were the engineering of nanofibers into a microporous structure and the use of human cells under non-animal and serum-free culture conditions, neither of which have been previously reported (44).

Kim et al., proposed an elliptical microporous array of nanofibers, dubbed the NOVA micropore array, with high AR and high pore density, which was not only capable of collecting cells in microwells with high cell seeding efficiency but also of producing multiple living and functional spheroids in a uniform and stable manner. Not only were human hepatocellular carcinoma (HepG2) cell spheroids cultured on the NOVA microwell array uniform in size and shape, but their viability was also enhanced. This facilitated the scalable production of a variety of living and functional spheroids and even organoids (45). Park et al., developed a process for fabricating nanofiber concave microvias (NCMs) with tunable size and shape. The use of a series of hemispherical convex electrolyte solution droplets as grounding collectors for electrospinning significantly improved the NCM’s degree of freedom in terms of size, shape, and curvature. Accordingly, researchers demonstrated the formation of spheroids from the human hepatoma cell line (HepG2) in NCM. Additionally, HepG2 cells were able to form spheroids that were homogeneous and of controlled size as a result of NCM (46). Thus, nanomaterials can be used to create novel micropores and thus generate novel organoid models, introducing a novel concept for organoid development.

### 2.3 Nanomaterials participate in the magnetic levitation culture of organoids

In 2010, Souza et al., reported a three-dimensional tissue culture based on cell magnetic levitation. In this study researchers injected magnetic iron oxide and gold nanoparticles into cancer cells and then magnetically suspended the cells in a liquid, thus performing cell culture (47). This is the first time that magnetic levitation technology has been utilized in the field of cell culture. In comparison to conventional culture methods, magnetic levitation culture allows for the manipulation of the geometry of cell masses and the clustering of multiple cell types in co-culture. Accordingly, Haisler et al., developed a comprehensive magnetic levitation method for 3D cell culture (48). Magnetic nanoparticle components consisting of gold nanoparticles, iron oxides, and cell adhesion peptide sequences were delivered to 2D cultured cells to make these cells magnetic, and then magnetism was used to control the cells, suspend the cells at the gas-liquid plane and generate extracellular matrix, and finally construct a 3D model. In general, magnetic levitation 3D tissue culture conforms to this culture method. Moreover, using magnetic levitation, Tseng et al., created 3D models, which were successfully used to construct adipose tissue organoids (fat globules) that preserve the heterogeneity of their constituent cell types *in vitro*. Correspondingly, researchers demonstrated the ability to assemble fat globules from diverse cell types, including adult stem cells (ASCs), endothelial cells, and white blood cells, which regenerate tissue. These fat globules mimicked the organogenesis of white adipose tissue (WAT) and were capable of forming vascular-like endothelial structures with lumens and monocular adipocyte differentiation. This established the foundation for high-throughput WAT culture and analysis (49).

**Figure 2:**
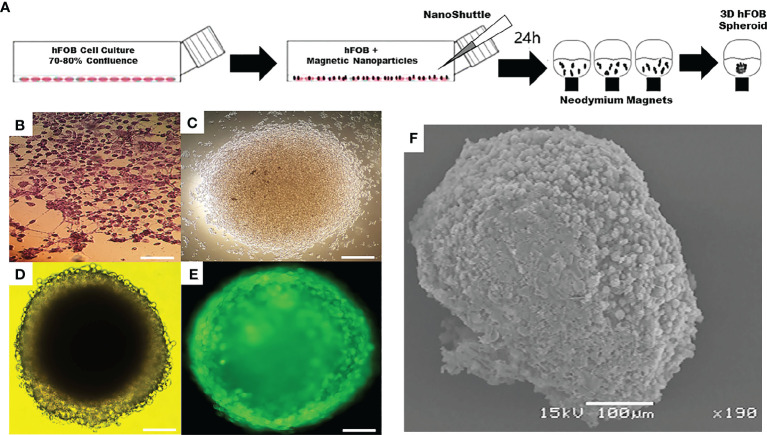
Application of magnetic levitation system in organoids. **(A)** Schematic of magnetic levitation technique. A confluent flask of hFOB cells was incubated with NanoShuttle overnight to allow for cell membrane-binding of the magnetic nanoparticles. The next day, the cells were seeded onto 96-well plate placed atop a magnetic drive of 96 neodymium magnets, the magnetic field influencing the hFOB cells to form an air-liquid interface and guide them to aggregate within hours of levitation to form the 3D Spheroid. **(B)** Optical micrograph of 2D tissue culture plate. **(C)** 3D hFOB spheroid after 3 h of magnetic levitation culture. **(D)** 3D hFOB spheroid after 24 h of magnetic levitation culture. **(E)** Fluorescence micrograph of 3D hFOB spheroid after 24 h of magnetic levitation culture. **(F)** Morphology of the 3D hFOB spheroid obtained by SEM with the presence of osteogenic factors incubated for 14 days under magnetic levitation system. FOB: fetal osteoblasts. Adapted with permission from (51), copyright 2021, Frontiers Media S.A.

In addition, Tseng et al., used a similar technique to create an organized three-dimensional (3D) bronchiolechial co-culture by layering cells sequentially to mimic natural tissue structures. The 3D co-culture model was assembled from four human cell types in bronchioles: endothelial cells, smooth muscle cells (SMCs), fibroblasts, and epithelial cells (EpiCs). Accordingly, this comprised the first attempt to combine these specialized cell types into an organized bronchiolechial co-culture. Magnetic levitation has been validated as a method for rapidly organizing 3D co-cultures, maintaining phenotype, and inducing extracellular matrix formation (50). Under magnetic levitation, Gaitán-Salvatella et al., were able to create 3D spheres of human fetal osteoblasts (hFOB) in their research. After 14 days of culture, the cell viability of 3D hFOB spheroids indicates that they are still viable. ALP assay, qPCR expression of Col1, ALP, and Itg-β1 molecules, and calcium deposition of alizarin red all demonstrated high levels of biological activity in 3D hFOB spheroids. In the presence of matrix deposition, SEM images allowed the morphological analysis of spheroids resembling 3D microtissues. These findings demonstrate that magnetic levitation culture can produce three-dimensionally stable osteoblast spheroids, and that the engineering application of bone tissue surgical regeneration in three-dimensional construction has a vast potential (**Figure 2**) (51). Bumpers et al., created for the first time nanomagnetic suspension 3D cultures of breast cancer (BC) and cancer (CRC) cells using carbon-coated cobalt magnetic nanoparticles, in which the suspended BC and CRC cells form microprotrusions. Suspension cultures have a high level of viability and persist for an extended period. In suspended 3D tumor spheres and xenografts of CRC and BC cells, the authors found that N-cadherin and epidermal growth factor receptor activity were highly expressed. Consequently, nanomagnetic levitation 3D cultures tend to form stable BC and CRC microtissues, which may be more applicable to a variety of applications in drug testing or regenerative medicine (52). Thus, in the current strategy for organoid magnetic levitation culture, nanomaterials are typically used to impart magnetic properties to cells, which are then suspended in the culture system by magnetic force, thereby enhancing the culture activity and maneuverability of cells.

### 2.4 Make bioreactors from nanomaterials and use them for organoid culture

Conventional techniques for organoid preparation are frequently reproducible and require expensive equipment. The bioreactor is a technical method that improves organoid reproducibility and homogeneity, and it can also promote organoid maturation (53). In recent years, there have also been reports of the use of nanomaterials in the construction of bioreactors for the production of novel organoids. Aalders et al., for instance, described a test method that permits the generation of functional cardiac micro-organs comprised of co-cultured cardiomyocytes and cardiac fibroblasts. Nanoparticles of hydrophobic fumed silica powder are used to encapsulate cells suspended in a drop of the medium. Nanoparticles treated with the hydrophobic chemical hexamethyldisilane (nHMDS) resulted in the formation of microbial reactors. These microenvironments were referred to as “liquid marbles” because they promoted cell coalescence and 3D aggregation. In addition, the nHMDS housing then facilitated optimal gas exchange between the liquid contained within and the surrounding environment. This microbial reactor was smaller and, therefore, suitable for higher throughput applications, making it an ideal co-culturing technique. Thus, the researchers demonstrated that the co-culture of cardiac fibroblasts and cardiomyocytes could be a valuable tool for simulating heart disease *in vitro* and evaluating cellular interactions to decipher disease mechanisms (54).

Brevini et al., described a protocol that permits the extraction of functional, pancreatic small organs from skin biopsies. The cells were suspended in a drop of medium and encased in hydrophobic polytetrafluoroethylene (PTFE) powder granules to create a “liquid marbles” microbial reactor that promoted cell coalescence and three-dimensional aggregation. In addition, the PTFE housing allowed for optimal gas exchange between the liquid inside and the surrounding environment. Additionally, it can reduce the size of experiments to work in smaller volumes, making it suitable for high-throughput applications (55). In these bioreactors, nanomaterials serve as encapsulation vessels, isolating the reaction system from the surrounding environment. Accordingly, new organoid culture techniques have great potential if other nanomaterial applications can be developed in bioreactors.

### 2.5 Nanoparticles involved in 3D bioprinting of organoids

Bioprinting is the use of computer-aided technology to pattern the printing of biological and non-living materials through designated 2D or 3D tissues in order to create bioengineered structures (56). The technology of 3D bioprinting is an extension of the technology of organoid culture. Inkjet bioprinting, extrusion bioprinting, and light-assisted bioprinting are common printing techniques (57). For instance, Urkasemsin et al., reported the use of magnetic 3D bioprinting (M3DB) to generate salivary adenoid epithelial organoids from stem cells. The neuronal network of these organoids responded to salivary nerve stimulants. This biological structure was created using a NanoShuttle-PL™ solution containing gold, iron oxide, and polyl-lysine (58). Bowser et al., utilized magnetic nanoparticles to create spinal cord spheroids in a three-dimensional hydrogel construct using magnetic bioprinting. The resulting structure exhibited local cell-cell interactions and long-distance projections that mimicked *in vivo* structures. Magnetic nanoparticles for spheroid formation provide batch-to-batch consistency in size and shape and reduce the need for skilled experimenters to place cultures precisely. This combined approach is a first step toward developing simple methods for integrating spheroids, hydrogel culture, and bioprinting as an alternative to more complex and costly procedures (59). In the study by Li et al., the researchers first 3D-printed a tubular composite scaffold capable of reconstructing bile duct function with real-time MRI imaging properties. Subsequently, then used ultra-small ultraparamagnetic iron oxide (USPIO) nanoparticles dispersed in gelatin methacryloyl (GelMA) as contrast agents to monitor the repair of lesion sites and degeneration of bile ducts in real-time MRI (60). In addition, bioinks combining the excellent shear-thinning properties of nanofiber cellulose (NFC) and the rapid crosslinking ability of sodium alginate were used for 3D bioprinting of human cartilage tissue and cells in the study by Markstedt et al (61).

### 2.6 Nanomaterials can give organoids more functions

**Figure 3:**
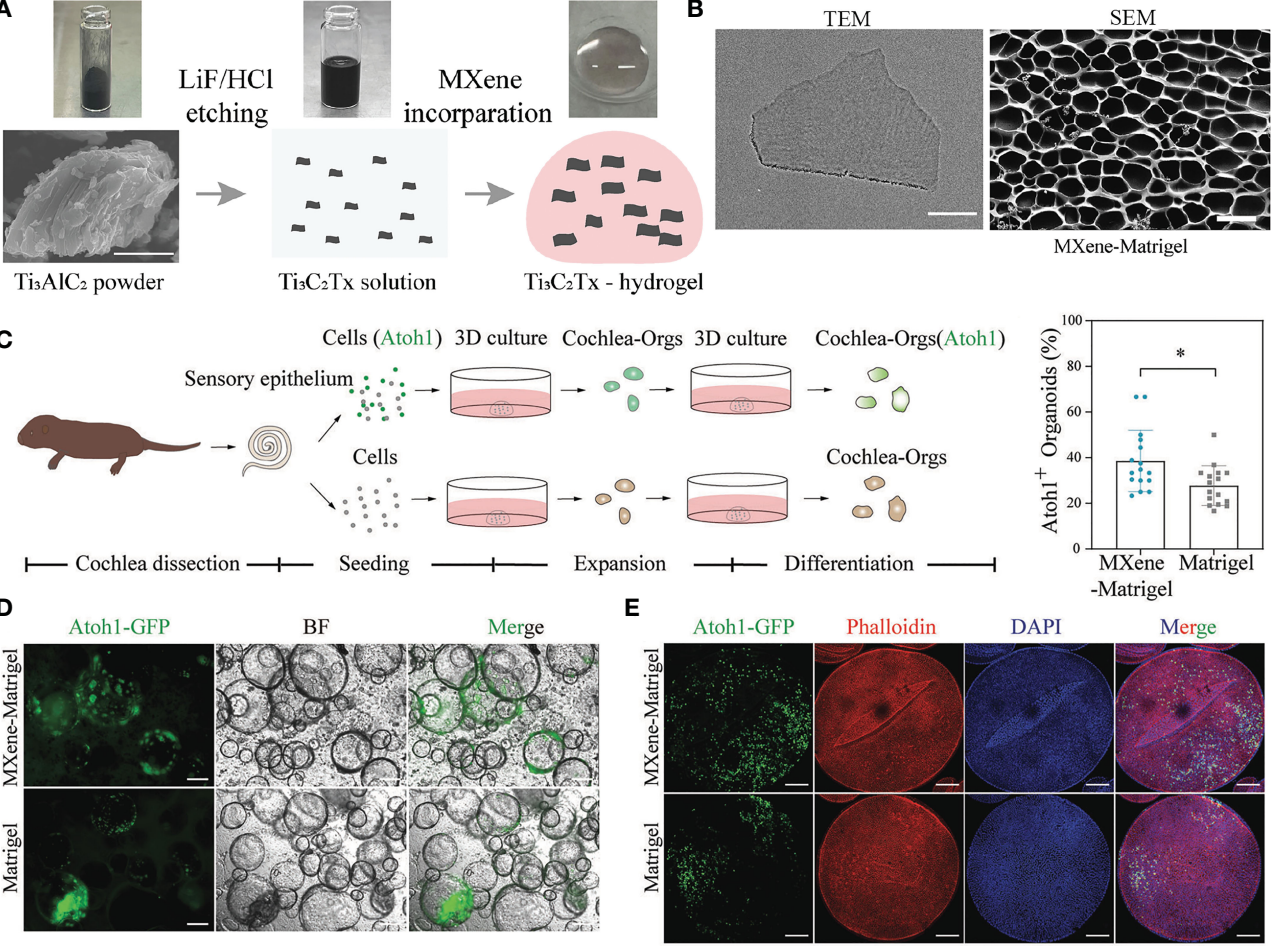
Ti_3_C_2_T_x_MXene-Matrigel hydrogel potentiated hair cells formation of Cochlea organoids. **(A)** Schematic diagram of the preparation of Ti_3_C_2_T_x_MXene-Matrigel. Appropriate amount of Ti_3_C_2_T_x_MXene solution was mixed with Matrigel, and the incorporating hydrogel was solidified at 37°C. **(B)** Representative TEM (bar: 200 nm) or SEM (50 μm) image of the Ti_3_C_2_T_x_MXene nanosheets. **(C)** Overview of the generation of cochlea hair cells through the differentiation of Cochlea organoids. **(D)** BF and green fluorescent (Atoh1-GFP) images of Cochlea-Orgs after 20 days of differentiation in the differentiationmedium. **(E)** Confocal images of DAPI (blue), early hair cell marker Atoh1-GFP, and phalloidin (red) (bar:100 μm). TEM, transmission electron microscope; SEM, scanning electron microscope; BF, bright field; GFP, green fluorescent protein; DAPI: 4’,6-diamidino-2-phenylindole. Adapted with permission from (62), copyright 2022, Wiley-VCH GmbH.

To date, no organoids have accurately represented their corresponding human organs. The development of new nanomaterials enables the creation of organoid culture systems that resemble human organs more closely. Adding nanomaterials to organoids or developing organoids using the properties of nanomaterials can increase the functionality of organoids, paving the way for future research. Zhang et al., for instance, incorporated Ti_3_C_2_T_x_MXene nanomaterials into Matrix in order to regulate Matrigel’s properties and demonstrated adequate biocompatibility. Ti_3_C_2_T_x_MXene Matrix (MXene Matrigel) controlled the development of cochlear organoids (cochlear tissue) by promoting the formation and maturation of organoid hair cells. In addition, the regenerated hair cells in MXene Matrix exhibited superior electrophysiological properties to those of Matrigel-regenerated hair cells. MXene Matrigel promotes hair cell differentiation by enhancing the mycin (mTOR) signaling pathway, whereas mTOR signaling inhibits hair cell differentiation. MXene Matrix also promotes synaptic formation efficiency and the establishment of innervation between regenerative hair cells grown from cochlear modiolus and helical ganglion neurons (SGNs) in co-culture systems. Accordingly, this method overcomes several limitations of the Matrigel-dependent culture system and significantly accelerates the application of nanomaterials in organoid development and hearing loss research (**Figure 3**) (62). Additionally, electrospinning was used by Beldjilali-Labro et al., to obtain poly(ϵ-caprolactone) nanofiber sheets, which were coated or uncoated with gold nanoparticles as a potential substrate for electrical stimulation. The differentiation of C2C12 cells was then measured over a seven-day period by the expression of specific genes and the confocal microscopy analysis of the arrangement and length of myotubes. It was demonstrated that multi-scale biological constructs possessed variable mechanical properties, supported skeletal muscle at different developmental stages, and improved the parallel orientation of the muscle tube with a variation of less than 15°. These scaffolds exhibited sustained myogenic differentiation by promoting the regeneration of skeletal muscle tissue (63). Moreover, Bao et al., investigated the beneficial effect of carbon nanotubes (CNTs) with different graphene layers and surface modifications on 3D models of intestinal organoids and demonstrated that CNTs promote the growth of intestinal organoids. Carbon nanotubes modify the metabolic profile of intestinal organoids and increase mitochondrial activity, respiration, and absorption of nutrients. These mechanisms promote the proliferation and differentiation of intestinal organoids through a synergistic effect. Thus, these results indicate that CNT has the potential to be used in intestinal tissue engineering (38). The main applications mentioned in this section are summarized in **Table 1**.

**Table 1:** Nanomaterials applied to assist in the construction of organoid 3D culture systems.

Methods	Nanomaterials	Organoid type	Functions	References
3D hydrogels	CNTs	Mice intestinal organoid	1. CNTs reduce the hardness of the extracellular matrix; 2. CNTs induce an increase in mitochondrial activity, accelerated cellular respiration and nutrient absorption.	(38)
	SiNP with 25–30 nm in diameter and 1 nm in thickness;obtained from Southern Clay Products Inc., USA	Mice B cell follicle organoid	SiNP increase the stability of the hydrogel system Making it closer to the stiffness of lymphatic tissue.	(39)
	BFP-1 laden MSNs (pep@MSNs)	Human bone organoid	pep@MSNs release BFP-1 to induce osteo-differentiation after cell spreading and expansion.	(40)
microwell array	Nanofibrous concave microwells (NCMs)	NA	Modify the properties of cell spheroids by controlling the shape of nanofibrous concave microwells.	(45)
	Nanofibrous scaffolds	Human parotid epithelial organoid	Increas efficiency of acinar-like organoid formation.	(43)
	Nanofibrous	Human hepatocellular carcinoma organoid	Enable microwell to possess both a high aspect ratio and a high well density	(44)
Magnetic levitation culture	Nanoshuttles (NS), consisting of gold, iron oxide, and poly-l-lysine;NanoShuttle (NS, Nano3D Biosciences, Houston, TX).	Murine adipose tissue organoids	By electrostatic attachment to the cell membrane, the cells are magnetized. When cells are resuspended in medium, they can be suspended from any stiff substrate by clumping them to the air liquid interface with a magnet placed above the culture vessel.	(48)
	NanoShuttle solution of magnetic nanoparticles (NanoShuttleTM-PL)	Human fetal osteoblast organoids	Electrostatically attach to the cell membranes and endowed the cells with magnetic properties	(50)
	A nanoparticle assembly consisting of poly-L-lysine (PLL),* magnetic iron oxide (MIO; Fe3O4, magnetite), and gold nanoparticles	Human bronchiole organoid	Biocompatible nanoparticles are taken up by cells and render them magnetic, allowing them to be magnetically manipulated	(49)
	C-Co nanoparticles	Breast cancer organoid	This nanomaterial can be used for internalization by cancer cells to achieve nanomagnetic suspension, and form three-dimensional cancer microtissues.	(51)
	Nanoshuttle (Greiner Bio-One, Monroe, NC)	Rat spinal cord organoid	Imparting magnetic properties to cells	(58)
	Hydrophobic fumed silica powder nanoparticles	Human cardiac organoid	The nanoparticles assit forming "liquid marbles" structures reducing the scale of the experiment. Thus, this technic enables higherthroughput applications	(53)
Bioreactor	NanoShuttle™-PL solution (Nano3D Biosciences, cat. no. 005-NS).	Human salivary gland organoid	Support cell proliferation and metabolism	(57)
	Hydrophobic polytetrafluoroethylene (PTFE) powder particles	Human pancreatic Mini-organoids	Support cell proliferation and metabolism	(54)
3D bioprinting	Nanofibrillated cellulose (NFC)	Human cartilage organoid	This nanomaterial composes a novel bioink with alginate. The novel bioink provids stability for 3D bioprinting of living cells at room temperature and atmospheric pressure.	(60)
	NanoShuttle™-PL	Human secretory epithelial organoid	support cell proliferation and metabolism	(57)
	Ultrasmall superparamagnetic iron oxide (USPIO) nanoparticles	Human bile duct organoid	Serve as the contrast agent to monitor the repair of the lesion site and the degradation of the bile duct in real time by magnetic resonance imaging (MRI)	(59)

## 3 The combined application of organoids and nanomaterials provides new strategies for disease research

As an emerging physiological model, organoids are applied increasingly in more and more research. Compared with traditional 2D models, organoids based on 3D culture technology and self-organization have characteristics that are closer to the original physiological morphology of organs. Organs are more suitable for research in organ development, drug research and development, and tumor treatment. In addition, organoids developed by patients’ own cells are expected to undergo autologous transplantation (64), avoiding the limitations of medical ethics. Using the patient’s own tumor cell culture organoids can provide personalized drug screening for patients, achieving precise medical treatment. Nanomaterial technology, as a rapidly changing research field, has been widely applied in various aspects of biomedicine. The chemical and physical properties of substances under nanostructures will undergo significant changes, and many therapeutic strategies have been developed. For example, the excellent optical properties of some nanomaterials have shown great application prospects in photothermal therapy (PTT) for cancer (65). The joint application between nanomaterials and organoids has also attracted the attention of researchers. Organoids can serve as models to verify the efficacy of targeted drugs based on nanomaterials and conduct drug screening; In addition, some nanoparticles can be used for live cell imaging and phenotypic analysis in organ like models; In photodynamic and photothermal therapy, researchers have developed many photosensitizers based on nanomaterials and tested them in organoids; The excellent properties of nanomaterials can also be used to develop electrochemical biosensors, which have been tested in organoid models in some studies. This article will discuss the joint application of organoids and nanomaterials in these aspects.

### 3.1 Drug screening

The 2D monolayer culture technique lacks a substance-signal connection within the organ; consequently, diseased cell types may lack disease-related input signals. In particular, the biological structure of organs, endogenous signaling, and cell-cell interactions may have a direct impact on the pathogenesis of disease. Therefore, 3D-cultured organoids that more closely resemble the physiological state of the human body have emerged as a model for drug testing. The majority of organoid-screenable drugs are chemotherapy drugs, small molecule-targeted drugs, and antibody drugs, among others. For instance, Zhang et al., conducted high-throughput drug screening on organoids derived from 40 patients with hepatocellular carcinoma (HCC) and determined that bortezomib (BTZ) was a highly cytotoxic small molecule against HCC. Using the flash nanocomposite/nanoprecipitation method, the researchers designed and manufactured sustained-release BTZ nanoparticles (BTZ-NP). BTZ-NP formulations demonstrated sustained BTZ release for 30 days. This BTZ-NP formulation was found to be highly effective at reducing tumor size and enhancing *in vivo* survival in three HCC animal models, including when administered via hepatic arteries (66). Kim et al., incorporated gold nanoparticles modified with hyaluronic acid (HA-AuNP) into a muscle bundle-based biohybrid robot that advances in response to electrical stimulation. HA-AuNP was incorporated into the fasciculus in order to increase its propulsion. Due to enhanced differentiation of HA-AuNPs and enhanced fascicular conductivity, the movement of the manufactured biohybrid robot was, therefore, enhanced. Moreover, the addition of positive and negative inotropic drugs produced dramatic motor changes in the manufactured biohybrid robot. Combining neural tissues such as motor neuron organoids and brain organoids, the proposed biohybrid robot demonstrated the potential to screen drugs for neuromuscular diseases (67). Le Joncour et al., described a protocol to obtain a hemo-cerebrospinal fluid barrier (BBTB) mimic by cultivating endothelial cells in contact with astrocytes on inserts at specific cell densities. In addition to evaluating tumor cell targeting in the same assay, this BBTB mimic can be used for quantitative and confocal imaging of nanoparticles crossing the endothelial and astrocyte barriers. In addition, the researchers demonstrated that the obtained data can be used to predict the behavior of nanoparticles in animal models used for preclinical research. This *in vitro* model can be adapted to other neurodegenerative diseases for determining the efficacy of new therapeutic molecules by BBBs and/or supplementation of brain organoids to assess drug efficacy directly (68).

### 3.2 Live cell imaging

**Figure 4:**
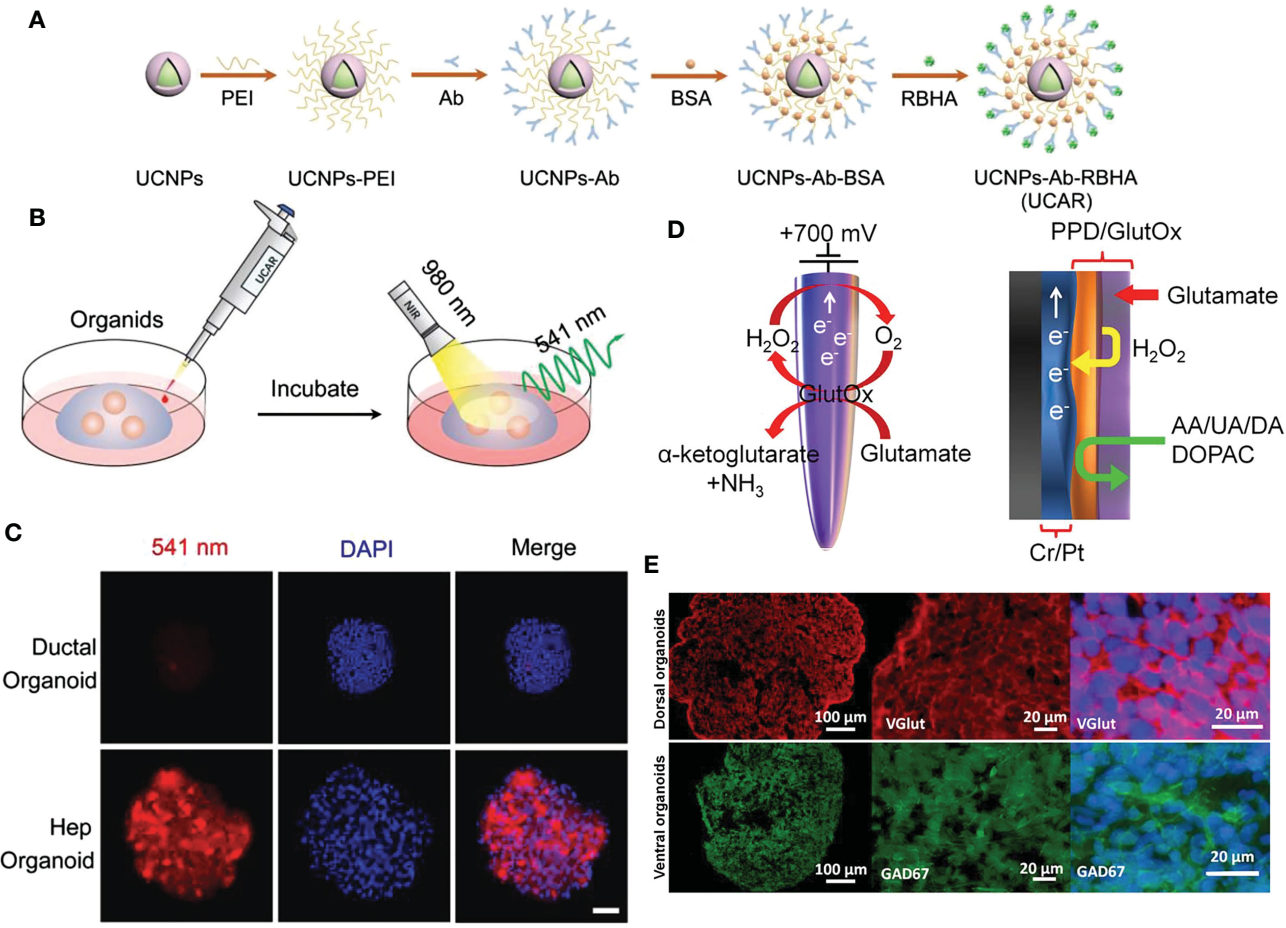
NMs for cell imaging and biosensor in organoids. **(A)** The schematic diagram of UCAR synthesis process from NaYF4:Yb3+/Er3+@NaYF4 (UCNPs). **(B)** The schematic illustration of albumin imaging in organoids using UCAR. **(C)** Ductal organoids and hepatocyte organoids were incubated with UCAR (red) for 3 h, followed by fixation and DAPI (blue) staining, and imaged by two-photon microscope under 980 nm excitation (bar: 50 μm). **(D)** Schematic representation of enzymatic reaction that allows detection of glutamate at the microelectrode, and graphical view showing PPD layer acting as a diffusion barrier to biomolecule species. H_2_O_2_ can reach the electrode while larger molecules are rejected. **(E)** Immunostaining images of hESC‐derived cortical dorsal forebrain organoids with strong expression of the glutamatergic marker, vGlut (red) and ventral forebrain organoids with expression of GABAergic neuronal marker, GAD67 (green). UCAR: upconversion nanoparticles, antibody, and rose bengal hexanoic acid; PPD, polypropylene diene monomer; hESC, human embryonic stem cell; GAD67, glutamate decarboxylase 67 kDa isoform. Adapted with permission from (69), copyright 2022, Wiley-VCH GmbH and (70), copyright 2018, Multidisciplinary Digital Publishing Institute.

Live-cell imaging refers to live-cell research utilizing time-lapse imaging technology; using live-cell imaging technology, the dynamic life processes involved in the target can be studied, and dynamic processes such as enzyme activity, signal transduction, protein and receptor transport, and membrane recycling process (endocytosis and exocytosis) can be detected. With the aid of live-cell imaging technology, scientists can observe the internal structure and physiological processes of cells in real-time or over time, thereby enhancing their understanding of cell operation processes. Liu et al., for instance, described *in vitro* luminescence methods for the detection of albumin, a marker of hepatocyte fate, and live-cell labeling with antibody (Ab) and rosean caproic acid (RBHA)-conjugated upconverted nanoparticles (UCNP). They used a “disconnect” strategy: In the presence of albumin, the transfer of energy to the quencher still inhibited the luminescence of the nanoparticles. Correspondingly, luminescence was restored following the albumin-antibody interaction under near-infrared light. UCNPs-Ab-RBHA (UCAR) nanoprobes have a broad detection range for albumin in a variety of biological samples. When applied to hepatic ductal organoid media, UCAR monitors hepatocyte differentiation in real-time by detecting secreted albumin. In addition, UCAR can image cytoalbumin in cells, organoids, and tissues in real-time. Accordingly, UCAR detected a decrease in albumin in liver tissue and serum in a CCl_4_-induced model of liver damage. Consequently, biocompatible nanoprobes with excellent stability and sensitivity are available for quantifying and imaging proteins in complex biological environments (**Figures 4 A–C**) (69). Balyasnikova et al., showed that fluorescent indocyanine lipids (ICL: DiD, DiI) formulated in polyethylene glycolated lipid nanoparticles (PLN) penetrate and accumulate efficiently in basement membrane (GBM). *In vitro* studies have demonstrated that PLN-formulated ICLs penetrate GBM spheroids and organoids more efficiently than liposomal ICLs. In the intracranial GBM model, more than 82% of the extravascular regions of tumors in the PLN group were fluorescence positive for ICL fluorescence 1 h after systemic injection, compared to 13% in the liposome group. In addition, PLN-formulated ICL accumulated in 95% of tumor myeloid suppressors and macrophages, 70% of tumor regulatory T cells, 50% of tumor-associated microglia, and 65% of non-immune cells 48 h after injection. Thus, the PLN-formulated ICLs were superior to pegylated liposomal doxorubicin and fluorescent dextran extravasation, and they accumulate in aggressive tumor margins and brain invaders more effectively. In contrast to liposomes, which are stable *in vitro* and *in vivo* in serum, PLN degraded prior to entering tumors, which may explain the disparity in their extravasation efficiency. These findings suggest an excellent opportunity to enhance therapeutic cargo delivery for invasive GBM (71).

Xie et al., developed a fluorescent probe for COX-2 imaging using a single-step procedure from rofecoxib. Using this novel strategy, six rofecoxib analogs were designed in total. Several analogs retained the relative COX-2-targeting activity of rofecoxib and also exhibited attractive fluorescent properties, which are studied experimentally and theoretically. Compared to Raw 264.7 cells expressing low levels of COX-2 and celecoxib-treated HeLa cells, the most potent analog 2a1, demonstrated strong COX-2 fluorescence imaging in HeLa cells overexpressing COX-2. Using brighter fluorescence in tissue sections or 3D organoids, 2a1 was able to differentiate between human cancer tissue and adjacent tissue. These findings demonstrate the potential of 2a1 as a near-infrared fluorescent COX-2 probe for clinical cancer imaging in humans (72). McCarthy et al., evaluated the ablation potential of CD44-targeted polymer nanoparticles utilizing hyaluronic acid (HA) as a targeting agent and coating it onto hybrid donor-acceptor polymer particles (HDAPPs) to form HA-HDAPPs using tumor organoid technology. In addition, only the photothermal polymer poly[4,4-bis(2-ethylhexyl)-cyclopente[2,1-b; Nanoparticles composed of 3,4-b’] was capable of producing nanoparticles composed of dithiophene-2,6-diyl-alt-2, 1,3-benzoselenodiazole-4,7-diyl] (PCPDTBSe) coated with HA to form HA-BSe NP. Monitoring nanoparticle transport in 3D organoids revealed a uniform diffusion of untargeted HDAPP compared to nanoparticle-matrix interaction-induced attenuated diffusion of HA-HDAPP. Calculating the diffusion curve suggests that HA-HDAPPs transport may be explained by diffusion alone, suggesting nanoparticle/cell-matrix interactions. In addition, photothermal activation revealed that only HA-BSe-NPs significantly reduced the viability of tumor cells in organoids. Although CD44-targeted therapy has limited transport of diagnostic nanoparticles, their targeted retention provides increased heat for enhanced photothermal ablation in 3D, thereby facilitating the evaluation of nanoparticle therapies prior to *in vivo* testing (73). Fang et al. designed the near-infrared small molecule fluorescent probe HD-Br with low toxicity and photostability for super-resolution imaging of lysosomes. Thus, while labeling lysosomes using the properties of the probe, lysosomal and mitochondrial interactions could be dynamically tracked. Due to the optimal near-infrared excitation and emission wavelengths of the probe, 3D imaging of liver organoids and imaging of Caenorhabditis elegans have been performed (74).

### 3.3 Photodynamic therapy

Photodynamic therapy (PDT) is a promising cancer treatment technology that employs a photosensitizer to irradiate a specific wavelength of light with targeted oxidative killing effects on diseased tissues to treat cancer while minimizing damage to normal tissues (75, 76). Nanomaterials have been commonly used to construct photosensitizer delivery systems and target transport to lesions; accordingly, certain nanomaterials can be used for PDT due to their exceptional fluorescence properties (77). On the contrary, organoids comprise an excellent PDT test bed. Therefore, the combined application of the two can provide researchers with the opportunity to develop new PDTs. By electroporating black phosphorus quantum dots (BPQD) into exosome carriers (EXO), Liu et al., were able to develop a photothermal agent that was highly effective. The resulting BPQDs@EXO nanospheres (BE) exhibited good biocompatibility, long cycle times, and excellent tumor targeting ability, thereby demonstrating remarkable photothermal therapy (PTT) efficiency via efficient tumor ablation *in vivo (* 78). Li et al., described a nanoassembled structure based on black phosphorus (BP) nanosheets and composed of cisplatin, BP, polydopamine (PDA), and hyaluronic acid (HA) for controlling cisplatin delivery, referred to as CBPH. In order to create CBPH, the surface of BP was double-modified by PDA and HA, which increased the stability, tumor-targeting ability, and photothermal efficiency of BP. Cisplatin is released in response to internal and external stimuli within the tumor microenvironment. *In vitro* experiments demonstrated that CBPH-treated 4 T1 cells exhibited an increased intracellular content of Pt and Pt-DNA adducts, which improved upon exposure to NIR light, resulting in potent antitumor effects via a synergistic mechanism (79). According to 2D monolayer and 3D organoid studies, the combination of CBPH and NIR phototreatment significantly inhibited the migration, invasion, and regenerative capacity of 4 T1 cells. This novel BP-based nanoassembly with controlled cisplatin tumor delivery and breast cancer metastasis inhibition broadened the application of BP in biomedical fields, thus holding great potential for future advancement (79).

Iqbal et al. used titanium dioxide-adsorbed Fe(iii) to create magnetic Fe-TiO(2) nanocomposites (NC), which played a role in achieving T(1)-weighted MRI contrast enhancement and enhancing the well-known photodynamic therapeutic efficacy of TiO(2) nanoparticles. Interestingly, the proposed NC demonstrated T(1) MRI contrast agent properties comparable to those of commercially available contrast agents. Moreover, the cytotoxicity induced by NCs in conventional methods is negligible and demonstrates significant support for the proliferation of intestinal organoids. It is anticipated that this research will serve as a guide for the development of additional biocompatible magnetic titanium dioxide-based nanosystems with multifaceted properties for biomedical applications (80). Obaid et al., described a (Cet, anti-EGFR mAb) photoimmune nanoconjugate (PIN) as well as *in vitro* and *in vivo* models of stroma-rich dyspancreatic ductal adenocarcinoma (PDAC) utilizing patient-derived pancreatic cancer-associated fibroblasts (PCAFs). In dystopic connective tissue proliferative tumors, Cet-PINs effectively penetrated blood vessels up to 470 μm, and photodynamic activation resulted in parenchymal tumor necrosis, which was not observed in T47D tumors (low EGFR) or when non-targeted constructs were utilized in both tumor types. Photodynamic activation of the Cet needle in dysproliferative tumors resulted in collagen photoregulation and a 1.5-fold decrease in collagen density, indicating that PDP may also be able to inhibit connective tissue formation. In addition, the *in vivo* safety of photodynamically activated Cet-PINs is significantly enhanced in comparison to non-targeted constructs. This is the first study to demonstrate the actual value of NIR-activated PIN-molecule targeting. This combined PIN platform and heterologous cell model paves the way for a wider range of multiplex combination therapies to synergistically control fibroproliferative tumor progression and extend PDAC patient survival (81).

### 3.4 New electrochemical biosensors

In electrochemical biosensors, the sensitivity of electroanalytical methods and the inherent bioselectivity of the biological component are combined. The biological component in the sensor recognizes its analyte, leading to a catalytic or binding event that ultimately generates an electrical signal monitored by the transducer that is proportional to the analyte concentration. Nanomaterials have exceptional chemical, physical, electrocatalytic, and other properties, in addition to their unique quantum size effects and surface effects, which are anticipated to further improve the performance of electrochemical sensing. Due to their stability, speed, accuracy, and low cost, nano-electrochemical biosensors have attracted a great deal of interest in the field of biomedicine and have made significant progress (82). In recent years, there has been an abundance of nanomaterial-based electrochemical sensors designed to detect specific physiological indicators of organoids for future research. For instance, Nasr et al., have developed a method to functionalize borosilicate glass capillaries with nanostructured textures as electrochemical biosensors to detect the release of glutamate by brain organoids produced by human embryonic stem cells (hESCs) that mimic different brain regions. For the oxidation of glutamate, biosensors exhibit obvious catalytic activity. Enzyme-modified microelectrodes can detect glutamate from 5 μM to 0.5 mM over a broad linear range. At various time points, measurements were performed on organoids, and results were obtained that were consistent. These findings demonstrate the biosensor’s dependability and utility for measuring glutamate concentrations over time in a single culture system (**Figures 4D, E**) (70).

Li et al., describe a procedure for the creation of cardiac cyborg organoids: First, the stretchable grid nanoelectronics are laminated onto continuous stromal sheets containing human induced pluripotent stem cells (hiPSCs) or progenitor cells derived from hiPSCs; subsequently, the cell pieces are aggregated into cell-dense plates by cell-cell attraction-induced cell proliferation and migration; and finally, the stretchable grid nanoelectronics are embedded in the cell plates and folded into tightly packed structures. The subsequent folding of the 2D cell plate/nanoelectron mixture into a 3D structure with a bowl-like geometry results in organ self-organization. Organogenesis unfolds densely packed nanoelectronics and distributes their structures throughout 3D organoids. Embedded three-dimensional nanoelectronics continuously monitor the electrophysiological behavior of stem cells and progenitor cells as they continue to develop and differentiate into various types of cells (83). A cyborg human brain organoid platform with “tissue-like” stretchable mesh nanoelectronics is described by Le Floch et al. By matching the mechanical properties of brain organoids and folding through the organogenesis process of stem cells or progenitor cells, stretchable electrode arrays can be distributed on 3D organoids. The tissue-integrated, stretchable electrode array does not impede brain organoid development, adapts to changes in volume and morphology during brain organoids, and maintains stable electrical contact with neurons within brain organoids throughout development. During early brain organoids development, electrodes coupled seamlessly and non-invasively to neurons allow long-term stable, continuous recording (84).

### 3.5 Immunotherapeutic studies combining nanomaterials with organoids

Last but not the least, organoids offer new opportunities for tumor immunotherapy. For example, Dijkstra et al. developed tumor organoids by resecting tumor specimens from patients with colorectal cancer (CRC) or doing core needle biopsies. Subsequently, the authors co-cultured tumor organoids and the patient’s peripheral blood to construct a ‘tumor organoid peripheral blood lymphocyte’ co-cultured model and obtained a population of tumor reactive T cells. These T cell populations kill tumor organoids and do not damage healthy tissue organoids, demonstrating that the generation of tumor specific T cells can be effectively induced by co-culture tumor organoids with immune cells, providing a new strategy for tumor immunotherapy (85). However, subject to the limitations of existing organoid structures and functions, organoid based tumor immunotherapy studies often require the participation of other regulators to mimic the complex tumor immune environment. Many studies have shown that nanomaterials exhibit excellent immunomodulatory effects (86). Therefore, combining nanomaterials and organoid technology might be a feasible strategy in the field of tumor immunotherapy. Q. Yin et al. reported a nanoparticle-based approach for immune environment modulation of tumor organoids. researchers formulated nanoparticles containing immunostimulatory substances that activated endogenous T cells in patient derived tumor organoids, and finally such endogenous T cells could exert inhibitory effects on tumor organoids (87). Zhang et al., using human brain organoids and glioblastoma co-cultured model to study the modulation of glial cells by dendritic polyglycerol sulfate (dPGS), demonstrated that dPGS has the effect of reducing inflammatory markers and glioblastoma invasiveness (88). Tumor immunotherapy research strategies of nanomaterials combined with organoids have not received much attention, and as a promising research direction, future investigators may gain more discoveries from them.

## 4 Outlook and conclusions

This article reviews the application status of NMs in various organoid culture systems and the application direction of NMs in combination with organoids in the biomedical field. Organoids and nanomaterials are two promising technologies that could revolutionize biomedical research. They can be combined to create personalized treatments, diagnostic and therapeutic devices, and various other medical instruments. However, researchers must also consider the magnitude of nanomaterials’ toxicity to human tissues; in this regard, organoids serve as a useful model (89). The organoid model can help verify the toxicity of nanomaterials without creating any ethical issues. Numerous studies have demonstrated that not all nanomaterials are non-toxic; Yu et al., for instance, investigated the intestinal toxic effects of graphene quantum dots (GQDs). Higher doses of OH-GQD caused significant intestinal damage, as evidenced by increased intestinal permeability, villi shortening, and crypt loss. Additionally, the authors used isolated crypts to establish three-dimensional organoid cultures, and the GQD treatment significantly reduced the size of surviving intestinal organoids (90). Hou et al. provided evidence of the toxic effects of plastic nanoparticles on the human intestinal system and explored the mechanisms involved (91). Likewise, the toxicity of some nanomaterials can affect the physiological properties of organoid models. In a study of brain organoids by Huang et al., it was demonstrated that silver nanoparticles inhibited brain organoid development and promoted apoptosis (92), showing neurodevelopmental toxicity. Therefore, the toxicity factors of nanomaterials should be taken into account in the development of organoids utilizing nanomaterials.

The application of nanomaterials in the field of organoids is not yet sufficiently advanced. In the studies mentioned in this article, the roles played by nanomaterials have assisted in the construction of cell scaffolds, the delivery of substances, the culture scaffold of cells and so on. Currently, no researchers have been able to use nanomaterials to overcome the limitations of organoid development, yielding landmark breakthrough results. Two reasons may have hindered the development of nanomaterials in the field of organoids: (1) existing nanomaterials technologies have not yet allowed the development of good enough biomaterials to be adapted to the organoid culture system; (2) the physiological and developmental landscape of the organ itself is poorly studied, leading researchers to fail to uncover critical culture factors and culture environments. But either factor, researchers will need more work into nanomaterials or organoids.

In conclusion, the use of nanomaterials can assist researchers in developing organoids that serve as suitable physiological models for disease research. In addition to reducing the duration and cost of drug development, the combination of the two can promote the creation of innovative medical technologies. Accordingly, the biomedical research applications of these two technologies appear to be limitless with further research and development.

## Author contributions

CS wrote the main text. Z-jZ designed, wrote the main text, and built the figures. Y-pH, Y-xW, and HZh collected the references. YW and LX reviewed the manuscript, and Z-tH and HZo revised the manuscript. All authors agree to be accountable for the content of the work. All authors contributed to the article and approved the submitted version.
